# What is the Tolerance Limit of the Human Society in Face of the Killing of Innocent and Defenseless People of Gaza? Stop the Killing of Children and Infants in Gaza

**DOI:** 10.34172/aim.2023.89

**Published:** 2023-11-01

**Authors:** Shahin Akhondzadeh

**Affiliations:** ^1^Roozbeh Hospital, Tehran University of Medical Sciences, Tehran, Iran

 The layers of ethics and human rights laws have been destroyed in the killing of the people of Gaza by the Zionist army. According to the basic rules of the Geneva Convention,^1^ attacking hospitals and medical centers is a major war crime. Without exception, all the hospitals in Gaza have been subjected to extensive bombardment, electricity and water cuts, and finally surrounded by tanks. Crimes not even committed by the Nazis in World War II, the Serbs in the attack on Bosnia and Herzegovina, or ISIS in their attack on Syria and Iraq.

 It is interesting to note that no United Nations organization, including the World Health Organization, UNICEF and even the World Red Cross, has taken any effective action so far. Suppose this genocide was carried out in a Western country, would the reactions be like this? What is a source of happiness is the clear conscience of the people of the world, especially in the Western countries, who, unlike their governments (demagogues on democracy and human rights) have stood united in support of the oppressed people of Gaza. I have no doubt that the brutal attack of the Zionist army on hospitals and the killing of more than 6000 children and infants will be included in the books and references of human rights and medical ethics as a war crime. As a person who has had the experience of being the head of a teaching hospital, I request all the heads of hospitals in the world to condemn the war crimes of the Zionist army and also the support of the Western governments for this crime. What is certain is that the Zionist government and the Western supporting governments in the Middle East have not sown wind this time but have sown a storm and they must know that they will reap a tsunami. Will we witness one day the leaders of the Zionists, like the leaders of the Nazis and the leaders of the Serbs, appear in the war crimes court and be convicted?
